# Dancing in time: feasibility and acceptability of a contemporary dance programme to modify risk factors for falling in community dwelling older adults

**DOI:** 10.1186/s12877-017-0476-6

**Published:** 2017-04-11

**Authors:** Laura Britten, Christine Addington, Sarah Astill

**Affiliations:** School of Biomedical Sciences, Faculty of Biological Sciences, University of Leeds, LS2 9JT Leeds, UK

**Keywords:** Older adults, Dance, Physical activity, Falls

## Abstract

**Background:**

Falls are a common cause of injury in older adults, with the prevention of falls being a priority for public health departments around the world. This study investigated the feasibility, and impact of an 8 week contemporary dance programme on modifiable physical (physical activity status, mobility, sedentary behaviour patterns) and psychosocial (depressive state, fear of falling) risk factors for falls.

**Methods:**

An uncontrolled ‘pre-post’ intervention design was used. Three groups of older (60 yrs.+) adults were recruited from local community groups to participate in a 3 separate, 8 week dance programmes. Each programme comprised two, 90 min dance classes per week. Quantitative measures of physical activity, sedentary behaviour, depression, mobility and fear of falling were measured at baseline (T1) and after 8 weeks of dance (T2). Weekly attendance was noted, and post-study qualitative work was conducted with participants in 3 separate focus groups. A combined thematic analysis of these data was conducted.

**Results:**

Of the 38 (Mean Age = 77.3 ± 8.4 yrs., 37 females) who attended the dance sessions, 22 (21 females; 1 male; mean age = 74.8, ±8.44) consented to be part of the study. Mean attendance was 14.6 (±2.6) sessions, and mean adherence was 84.3% (±17). Significant increases in moderate and vigorous physical activity were noted, with a significant decrease in sitting time over the weekdays (*p* < 0.05). Statistically significant decreases in the mean Geriatric Depression Scale (*p* < 0.05) and fear of falling (*p* < 0.005) score were noted, and the time taken to complete the TUG test decreased significantly from 10.1 s to 7.7 s over the 8 weeks (*p* < 0.005). Themes from the focus groups included the dance programme as a means of being active, health Benefits, and dance-related barriers and facilitators.

**Conclusions:**

The recruitment of older adults, good adherence and favourability across all three sites indicate that a dance programme is feasible as an intervention, but this may be limited to females only. Contemporary dance has the potential to positively affect the physical activity, sitting behaviour, falls related efficacy, mobility and incidence of depression in older females which could reduce their incidence of falls. An adequately powered study with control groups are required to test this intervention further.

## Background

Falling is a common cause of serious injuries in community-dwelling older adults [1] and poor outcomes following falls are exacerbated by the numerous co-morbidities prevalent in an older population. Due to both the economic, social and humanistic cost associated with falls, the prevention of injury associated with falls in older people is a public health target in many countries around the world [2].

Falls are multifactorial in nature, however modifiable risk factors that predispose an individual to fall have been identified, which present opportunities to implement interventions designed to reduce falls and improve successful aging [3]. Intrinsic modifiable risk factors are categorised as either physical or psychosocial. Physical factors include reduced physical activity [4–6], muscle weakness [7], and deficits in gait and/or balance [8, 9]. While not as extensively investigated, psychosocial factors include depression [10, 11] and cognitive impairment [12]. More recently, fear of falling or fall-related self-efficacy has also been identified as a risk factor for falling [2], particularly when it results in avoidance activities, and causes a decrease in physical activity [13], and overall health related quality of life [14].

A recent Cochrane systematic review concluded that physical activity and exercise programs (containing components of strength, balance and flexibility) are one of the single, most effective strategies to reduce the rate of falls in community dwelling older adults [15]. Overall, group exercise classes tend to be more effective at fall prevention, and Tai Chi also seems to reduce the risk of falling [16]. Dance shares many similar qualities to Tai Chi [17], and is classified as a ‘3D’ exercise, as it involves ‘constant movement in a controlled, fluid, repetitive way through all 3 spatial planes’ (Prevention of Falls Network Europe, 2007, p. 21). Dance is likely to be a more familiar concept than Tai Chi to the current aging population as it was very much part of life in the early and mid-twentieth century [18], and is a popular recreational activity utilised during health interventions for individuals of an advanced age [19].

Over the past 10 years the number of dance- based studies involving older adults have increased in number, supporting the benefits of dance in improving a range of physical functions [20, 21]. For example Hopkins et al., [22] showed that dancing improved cardio-respiratory endurance, agility, flexibility, balance and lower limb strength, the latter being strong risk factors for falling. Eyigor et al., [23] observed that an 8 week folklore dance intervention significantly improved physical performance (e.g. balance, endurance), while Hui et al., [24] reported 12 weeks (2 sessions a week) of low-impact aerobic dance, improved dynamic balance and mobility in the time up-and-go. The effect of dance on psychosocial functioning is less well established. Eyigor et al., [23] showed dance to improve aspects of quality of life (e.g. mental health) in older women, while Murrock & Graor, [25] and Jeon et al., [26] reported a decrease in depression.

Overall, the above data suggest that dance could comprise one strategy to reduce falls in community dwelling adults. However, Haboush et al., [27] noted that older people may experience ballroom and aerobic dances as very frustrating as they fail to ‘learn’ the sequence of steps needed. In contrast, social dancing encourages a playful, spontaneous atmosphere, affording an opportunity to reconnect with one’s memory, youth, and history. Roberson & Peclova [28] and Nadasen [29] also noted the social advantages of taking part in dance as a group, noting that the effects of social dance went beyond the physical and also improved both overall well-being and quality of life.

One area of dancing that has been relatively understudied is contemporary dance. Contemporary dance in community settings can be a low-physical impact activity that is therefore open to all regardless of their baseline physical condition skills or ability [30]. The Arts Council England (2009) define contemporary dance ‘dance that is contemporaneous, i.e. that is made today, which offers an insight into the world and its emotion, interaction and behaviour through the language and its relationship both with itself and others’ [31]. Contemporary dance offers the opportunity to improvise or interpret the music or feelings at a more person centred level, individually or as part of a larger group through movement which includes elements of aerobic exercise, balance activities, low-level resistance exercise, and moves to enhance flexibility [32]. Coubard et al., [33] showed that approximately 6 months of contemporary dance improved the ability to switch attention between different tasks, while a falls prevention programme or a Tai Chi Tuan programme yielded no such positive effects. Improvements in the ability to switch attention have been suggested have a significant impact on reducing fall-risk as a result of a more efficient allocation of attentional resources which subsequently reduces the fear of falling [34]. In a second study, Coubard et al., [35] also demonstrated that 1 month of contemporary dance benefited postural control of older adults, another modifiable risk factor for falls.

Overall, published work to date shows that dance has the potential to minimise risk of falling through the modification of risk factors such as fear of falling but also due to improvement in other physical (e.g. changes in physical activity levels) and psychosocial factors (e.g. depression). However, no one study has examined the efficacy of CD to do so. In this study we developed a small scale pilot study to examine the effect of an 8 week contemporary dance programme on both physical (e.g. physical activity, balance, mobility) and psychosocial (fear of falling, depression) risk factors for falls [2]. Our intervention was designed in line with the U.K. Medical Research Council guidelines for complex interventions [36]. These argue for the importance of pilot work to investigate the feasibility of implementing and assessing the intervention as intended. The intervention acceptability and feasibility was addressed by documenting attrition and adherence rates, in addition to changes in physical and psychosocial risk factors for falls. Furthermore, we used focus groups to document participants’ views of the intervention and how it had affected them.

## Methods

The intervention was co designed by 4 groups of experts; (1) Sport and Exercise Scientists (2) Dance artists (3) Public Health Experts; specialising in older adults and falls prevention (4) an independent panel comprising community dwelling adults aged 60–85 years of age. The intervention comprised an 8 week dance programme delivered by dance choreographers in local community facilities across 3 sites in West Yorkshire.

### Participants and study design

An uncontrolled (pre-post) intervention design was used, with 3 independent samples. Data was collected at baseline (after 3 trial dance sessions, T1) and after 8 weeks of dance (T2). All study procedures were undertaken by 2 members of academic staff from the University of Leeds. All data were collected in local community facilities at the end of the dance session, and collection was staggered at each site over a 1 year period. Participants were permitted 3 taster sessions (deemed essential by the older adults who contributed to the design of the dance programme) and then were invited to take part in the research. Thus baseline data were collected in session 4 (T1) and post intervention data were collected in session 20 after 8 weeks of dancing, or 17, 90 min sessions (T2). Participants completed a series of questionnaires, performed the Timed up and go test at T1 and T2, and were also invited to participate in a focus group that discussed attitudes towards the intervention and barriers and facilitators to continued participation.

Neighbourhood networks who were based in the most deprived wards in Leeds were invited to take part. These groups were purposefully selected as they lived in areas of Leeds with the greatest health inequalities. Furthermore, it was anticipated that they would have high rates of physical inactivity, and will thus derive the most benefit from the intervention. A representative from the neighbourhood network worked with the research team to recruit participants. This was considered to be important as the representative was known to potential participants as an important part of an existing community group, and also acted as a trusted person, independent of the research team that older adults could discuss the nature of the dance and the research element with. To be considered for inclusion participants had to be 60 years of age or more, had to be able to speak and understand English, live independently, were able to attend dance classes in a pre-arranged community facility, and were not currently part of a falls prevention programme following a fall. Participants were not excluded from the study based upon their physical ability to perform dance as, the nature of contemporary dance ensures that the sessions could be adapted to suit all. Eligibility was based on participant self-report only; no further screening (e.g. physical health) took place. Due to the nature of recruitment refusal rates could not be calculated, and due to ethical reasons participants could take part in the dance programme without having to be part of the research study.

### Contemporary dance intervention

The structure of the sessions was always organic and fluid which represented the continual responsiveness of the artist to the needs and agency of group and towards individuals. The general structure of each session however was as follows: (1) The session opened with a warm up to prepare the body for dance through orientation to space or others and a conversation between the artists and participants. (2) Individuals then moved their body using active and passive movements of all joints, and basic low impact aerobic movements e.g. walking on the spot or while seated. (3a) Following this, individuals were introduced to a series of moves that were practiced to music either individually or in groups as a sequence, the latter being important to create a sense of collectively, and encourage tactile interaction (3b) Improvisation. The dance artist proposed a theme e.g. an action, object or word and individuals were asked to explore that theme on their own and then taking into account others presence, or by interacting with another individual. The time allocated to 3a and 3b varied, with time spent completing dance in 3b being very much informed by the dance artist leading the intervention. (4) Cool down. Participants cooled down using deep breaths and passive and active stretching. Participants were offered the opportunity to talk and partake in refreshments at the end of the session (20–30 min). At the end of the program participants invited friends, neighbours, and family to the community facility to ‘share’ their dance with them. This ‘sharing’ was not often a piece of choreography or a product but was instead a sharing of the processes, tasks and a mixture of specific moves and improvisations to music. There was no cost to the participants to attend the sessions. Prior to the beginning of the dance intervention, the instructors completed a 1 day workshop that prepared them for dancing with the older adult. The workshop was ran by a dance artist who was experienced in working with older adults in a contemporary dance setting. The course consisted of a sharing of experiences and expertise in delivering dance activities to older adults, how to adapt movements to suit different needs and discussions around the kinds of barriers that older adults may find during physical exercise.

## Outcomes

### Quantitative data

The only demographic data that were recorded was age, gender, and health status. All were self-reported at T1 only. Attendance at each session was noted to allow calculation of adherence to the programme.

### Physical activity and sedentary behaviour

The IPAQ (International Physical Activity Questionnaire) was used to assess physical activity and sitting time or sedentary behaviour [37, 38]. The IPAQ assessed sitting time during the week and weekend in addition to the total time over the preceding seven days. Items also assessed how much time was related to walking or in moderate or vigorous physical activity respectively over the previous seven days. Vigorous activities were defined as those that take physical hard effort and make you breathe more than normal, and moderate as those that take moderate physical effort and make you breathe somewhat harder than normal. For both of these items participants were asked to consider only those physical activities that you did for at least 10 min at a time and responses to all IPAQ measures were provided in hours and minutes, and converted to total minutes for the purpose of analyses.

### Timed up and go

The timed up and go (TUG) was used to assess mobility [39]. The TUG is recommended as a routine screening test for falls in guidelines published by the American Geriatric Society and the British Geriatric Society [40], and The National Institute of Clinical Evidence (NICE) guidelines also advocate the use the TUG for assessment of gait and balance in the prevention of falls in older people [41]. Participants are asked to stand up from a chair, walk for 3 m, turn around, walk back and sit down. The participants were asked to complete this as quickly as possible, while being as comfortable and as safe as possible, and it was timed with a stopwatch to the nearest 0.1 s [42].

### Falls efficacy scale-international (FES-I)

Falls self-efficacy encompasses fear of falling, self-efficacy, balance-confidence and activity limitation and is defined by the ProFANE working group as ‘the degree of confidence a person has in performing common activities of daily living without falling’ [2]. The FES-I has excellent psychometric properties [43, 44] and is better suited to studies that wish to examine the effect of ‘treatment’ on fear of falling, and is slightly more sensitive to change [45]. To obtain a score for the FES-I, all scores from each item are added together to give a total that will range from 16 (no concern about falling) to 40 (severe concern about falling).

### Geriatric depression scale

The Geriatric Depression Scale (GDS) short form evaluates signs of depression among older adults [46]. It comprises a series of 15 questions with yes and no answers, with each being allocated a 0 or a 1 according to the published scoring convention. The maximum score is 15 and minimum score 0.

### Qualitative data

Focus groups were chosen as a method of data collection. Focus groups facilitate development of thoughts and ideas through participant interaction in a comfortable, safe and supportive environment [47]. One focus group was held at each site, with 6–9 participants in each focus group. Each focus group lasted 35–50 min and was digitally recorded. The focus groups had a semi-structured design with follow-up probes on key topics of interest. Member checking was undertaken both formally and informally as opportunities arose during the focus group [48]. Questions were developed and piloted with the independent panels who contributed to the overall study design. The focus group questioned (1) experiences of the dances programme (2) perceptions of how the dance had affected the participants e.g. physically, socially etc. (3) facilitators and barriers to participation in dance programme.

## Data analyses

### Quantitative data

We used appropriate descriptive statistics (frequencies, percentages, means and standard deviations) to describe the sample, and measures of recruitment and adherence to the study. Mean adherence to the dance programme by all participants was calculated by noting each session that was attended and then dividing this by 17 the total number of session the study covered. For example, if a participants attended 13/17 sessions their adherence would be 76.5%. The changes in physical activity, sitting behaviour, depression, mobility and fear of falling were assessed using paired samples t-tests, an alpha level of 0.05 was adopted for all analyses.

### Qualitative data

All focus group and interview recordings were transcribed verbatim and anonymised. Data analyses used a thematic content analysis, which consists of interpreting meaning from the context of the textual data to enable a better understanding of the phenomenon under investigation [49]. Themes were inductively developed and iteratively refined by one coder (SA), and verified through discussions with a second coder (LB). Disagreements were resolved through discussion between coders.

To identify final themes we followed the recommendations of Braun & Clarke [49]. First, a combination of these and candidate themes were identified. The transcripts were read line by line and the text marked with code/s that described the content of the response. Then, we- refocused the analysis at the level of a theme, both candidate and sub-theme. This involved sorting the different codes into potential themes, and collating all the relevant coded data extracts within the identified themes. From this, the coded data and candidate themes were reviewed, and final themes emerged. As a result of this iterative process, overarching themes across the three sites were identified. Potential quotes that were deemed to best represent the nature of each theme were then extracted, discussed by the authors and a final selection of quotes produced.

## Results

### Quantitative data

#### Sample description and adherence rates

A total of 45 older adults initially joined the programme, but within the first 3 sessions, 7 (15%) dropped out (2 males) citing lack of interest or and a further 5 (females) due to conflict with other activities. A total of 38 older adults (Mean Age = 77.3 ± 8.4 yrs) completed the dance programme, all were female except 1 male at site 2. Of these 38, only 22 (21 females) consented to complete the research element of the dance programme. The mean age of this sample was 74.8 ± 8.44 years, and a mean number of 14.6 (±2.6) sessions were attended and the mean global adherence rate was 84.3 ± 17.03%. Descriptive data relating to each site sample are presented in Table 1, but due to small sample sizes statistical analyses was not completed.

**Table 1:** Participant characteristics for each site sample

Participant characteristics		Site 1 Research (*n* = 11)	Site 2 Research (*n* = 9)	Site 3 Research (*n* = 4)
Gender (n)	Male	0	1	0
	Female	11	8	4
Age (years)	(mean, SD)	70.9 (8.1)	76.3 (12.1)	75.7 (7.4)
Adherence (% sessions attended)	(mean, SD)	82 (15)	88 (11)	85 (23)
Number of session attended.	(mean, SD)	14(3)	15 (2)	15 (4)

#### Behavioural responses to dance programme

**Fig. 1:**
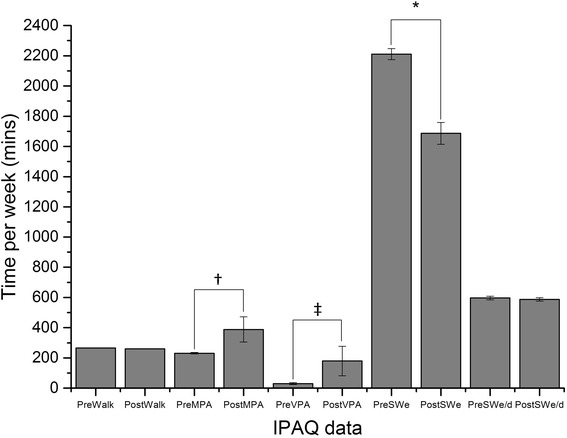
Mean pre and post values (±standard error) from the International Physical Activity Questionnaire for minutes per week spent walking, carrying out moderate physical activity (MPA), vigorous physical activity (VPA) and sitting during the week (SWe) and weekend (SWe/d). (* denotes significance at *p* < 0.05, † reflects significance at *p* < 0.01 and ‡ represents a significant difference at *p* < 0.005)

Table 2 shows the trends in behavioural responses at T1 and T2 across all three sites. However, statistical analysis was performed on the sample as a whole due to low sample sizes at each site. As Fig. 1 indicates, walking, moderate and vigorous physical activity increased between baseline and the end of the dance programme, however, only moderate physical activity levels (t (21) = 2.19, *p* < 0.05) and vigorous physical activity (t(21) = 2.59, *p* < 0.005) increased significantly. Fig. 1 also indicates, that sitting time decreased during both the weekday and at weekends, however, only the former decreased significantly (t(21) = 1.81, *p* < 0.05 *d* = 0.20); (see Table 3 for analysis of the number of participants per site increasing physical activity or decreasing sitting behaviour.

**Table 2:** Sedentary Behaviour, Physical Activity, and Behavioural Response data at T1 and T2 and Sites 1–3

	Site 1*		Site 2*		Site 3*		Overall	
	T1 (Mean, SD)	T2 (mean, SD)	T1 (Mean, SD)	T2 (mean, SD)	T1 (Mean, SD)	T2 (mean, SD)	T1 (mean, SD)	T2 (mean, SD)
Sedentary behaviour								
Sitting time weekday (mins/week)	2333.0 (481.6)	2170.5 (429.5)	2515.5 (631.4)	2545.0 (848.6)	1785.0 (820.3)	1570.0 (172.3)	2299.5 (713.4)	2197.3** (713.4)
Sitting time weekend (mins/week)	762.0 (80.3)	762.5 (87.7)	682.5 (166.5)	655.3 (176.2)	345.0 (179.2)	650.0 (179.2)	720.0 (134.7)	702.7 (134.7)
Physical activity								
Walking (mins/week)	294.8 (170.0)	284.5 (191.0)	324.4 (185.6)	315.6 (176.3)	180.0 (176.6)	180.0 (176.6)	284.3 (176.3)	276.6 (181.2)
Moderate (mins/week)	191.6 (151.6)	278.5 (171.0)	358.8 (149.1)	358.8 (149.1)	142.5 (189.4)	142.5 (189.5)	243.2 (175.2)	305.7** (217.9)
Vigorous (mins/week)	34.0 (58.9)	50.0 (70.1)	47.5 (75.5)	47.5 (75.5)	10.0 (20.0)	435.0 (370.0)	34.6 (60.2)	51.8** (70.6)
Behavioural Responses								
Timed Up and Go (seconds)	9.6 (4.3)	7.6 (1.0)	11.0 (3.2)	7.4 (1.0)	8.5 (5.6)	7.7 (4.4)	10.1 (4.2)	7.7 (2.8)***
Geriatric Depression Scale	2.1 (3.1)	1.1 (1.4)	5.3 (3.7)	3.5 (4.32)	2.3 (2.22)	2.0 (1.83)	3.2 (3.3)	2.1 (2.8)**
Falls Efficacy Scale	24.1 (8.5)	19.4 (4.3)	28.7 (9.5)	23.8 (6.2)	32.6 (12.2)	29.0 (16.4)	27.6 (9.9)	23.7 (8.6)**

*Site 1 IPAQ N=11, TUG=8, FES-I=9, GDS=9; Site 2 IPAQ N=9, TUG=5 FES-I=7, GDS=6; Site 3 IPAQ N=4, TUG=4, FES-I=4 GDS=4.

** Indicates a significantly different mean score at T2 compared to T1, *p* <0.05, or ***p <0.005

**Table 3:** Absolute and Relative Changes in Sedentary Behaviour, Physical Activity, and Behavioural Responses

	Increase N (%)	No Change N (%)	Decrease N (%)
Sedentary Behaviour			
Sitting time weekday (IPAQ) (mins/week)	1 (4.5%)	14 (63.7%)	7 (31.8%)
Sitting time weekend (IPAQ) (mins/week)	3 (13.6%)	13 (59.1%)	6 (22.3%)
Total sitting time (IPAQ) (mins/week)	2 (9.1%)	11 (50%)	9 (40.9%)
Physical Activity			
Walking (IPAQ) (mins/week)	10 (45.5%)	9 (40.9%)	3 (13.6%)
Moderate physical activity (IPAQ) (mins/week)	13 (59.1%)	5 (22.7%)	4 (18.2%)
Vigorous physical activity (IPAQ)(mins/week)	4 (18.2%)	18 (81.8%)	0 (0%)
Behavioural Responses			
Timed Up and Go (seconds)	0 (0%)	3 (16.6%)	14 (82.4%)
Geriatric Depression Scale	2 (10.5%)	11 (57.9%)	6 (31.6%)
Falls Efficacy Scale	1 (5%)	6 (30%)	13 (65%)

Note: All sedentary behaviours and behavioural responses, a decrease denotes an improvement on this measure, for all physical activity measures an increase denotes an improvement

Table 2 also shows a statistically significant improvement with respect to scores on the GDS (t(18) = 1.99, *p* < 0.05), and FES-1 (t(19) = 3.55, *p* < 0.050) and a statistically significant decrease in the time taken to complete the TUG test (t(16) = 4.46, *p* < 0.005). It should be noted that some participants felt uncomfortable answering questions on the GDS, and no participants refused to complete the TUG and FES-1.

### Qualitative responses to the dance programme

Three overarching themes were extracted; the dance programme as a means of being active, health benefits and dance-related barriers and facilitators. Unless otherwise stated, all points apply to participants from the three focus groups.

### The dance programme as a means of being active

The intervention was viewed positively by the participants and reminded them of the need to be active:

*‘My main reason for doing it is because I quite enjoy it and it get me out from sitting down and sleeping and I get to meet- and meet people while exercising, it was nicer to, you know exercise like this…..its good for me’.*

*‘ ….i use a walking frame and I’m trying to keep my legs and body active, so that’s why i come….it was different, to you know, get out and do exercise in this way…..’*

Others noted it as a new way of exercising, that was either complementary to their existing activity or more often as a new activity that was different to current opportunities.

*‘Well I do two sessions of different activities a week so this was just something to add on to those something that was a bit, you know, different…and its got to be good for you hasn’t it, moving’.*

‘ I *think this dance class is lovely. I didn’expect …. that it was more like…. Ballroom dance or things like those, but it‘s not really dance to me, its more like exercise you know. Well it’s a mixture….i could express myself more and do a little more here among people that i wouldn’t do at home’.*

In addition, the group nature of the exercise led to a sense of obligation to attend and be active. Participants felt that they did not want to let each other down, so it was important to attend each session, and it was more enjoyable than exercising on your own:

*‘you do it because you do it in groups…’ ‘…you're letting the other people down aren't you if you are not here? Its nicer us as a group, I just sit on my own otherwise…’.*

### Health benefits

Within this main theme, participants noted both physical and psychological benefits they attributed to the dance intervention. Within physical benefits participants noted reductions in pain, stiffness in joints, increased energy levels, balance and coordination.

*‘… the emphasis doesn’t seem to be as much on the dance…as your body, the things you can do with your body, the stretching and you know the coordination and balance, which is exactly what I needed. I feel better about; you know bending or moving about when shopping…’.*

Secondly other comments were grouped under the subtheme of psychological benefits. For example, participants noted on how they felt they had used their brain, in addition to just being physical:

‘*i think the thing is we’ve actually used our brain to put the exercise that we’ve done into a little routine…..a little dance… so it’s made it so we’ve used our brain as well, as well as us body…….when I go home I like to practice, in my head, to get my brain working…’.*

In addition, participants also noted positive affects on their quality of life and their mental health:

*‘Well its great here, been a lifeline. I mean if I’d have been in xxxx I’d have been lonely, i think its part of mental health as well as physical it helping. I mean we all have a good laugh, we all go home feeling good don’t we? Yeah, and like we’ve all gelled really well haven’t we?’*

### Dance-related barriers and facilitators

Physical barriers included existing health conditions. For example, some participants reported that they were unable to perform some dance moves due to pre-existing health conditions, but many reported that the instructor had adapted the movement to their personal circumstances.

‘….*Yeah it was lovely, that’s how I feel about here, you know, you can’t….you can’t stand- oh well I cant stand all the time and to know you can still dance with somebody, well I sit and we get on with it*.’

Participants stated that location was important and centres in the community were preferred locations, this was considered a logistical barrier and facilitator.

*‘Here, yeah we like it here, yeah its only five doors away from me. It’s local, others can walk down, yeah locality is really important …anywhere else it’s awkward for some, isn’t it?’*

Along with accessibility, financial cost was identified as a logistical barrier to taking part in the programme as travelling to and from classes was considered expensive. One participant said:

*‘I suppose costs can be a problem, - sometimes if you have to pay on a bus, you’ve got to consider people’s journey and….appropriate timing. Obviously the cost of it-if it’s-there is a cost to it. If you’re doing a lot of other tings that are costing so…..because some of the exercise classes can be quite expensive’.*

The one aspect of the intervention that the participants suggested more thought was given to was the balance of creative vs more didactic dance, although the creative dance was viewed positively for aiding interacting with people that you didn’t know:

*‘I think id like to a more exercise routine and less creative. It’s a novelty for us. I think its rather too big a helping for some of us, of being creative’.*

*“…because if you feel shy about approaching somebody. If you actually dance with them it makes such a difference, because you are not looking at each other in a way you might have, yes the improvising and the playing together, playing for adults that’s what it is’.*

Overall, participants reported that they would be happy with a similar programme in the future. One suggestion that arose was the prospect of taking home something, beyond the music, to help them practice their dance steps:

‘ *I take the dance home with me, many times when I’m home and I do the dancing, then its coming into my head. But I cannot remember it all….i do the dancing, either sometime in bed or if I put the tape on and get out and do a little, because we had a piece that we thought we would do Monday and I was trying to get that …..i like to practice but need something to help, except the music…’.*

## Discussion

This study explored the feasibility and acceptability of a contemporary dance programme to modify physical and psychosocial risk factors for falls in community dwelling older adults. This was completed using four sources of information: rates of attrition and adherence, pre and post changes in behavioural responses to the intervention, and reflections on the intervention expressed during focus groups.

The main findings were that over the 20 sessions, the attrition rate was 15%, with an overall mean adherence rate of 84.3%. Analyses of the behavioural response data showed there was a decrease in sedentary or sitting behaviour on weekdays, and an increase in moderate and vigorous physical activity patterns (Fig 1). Furthermore, significant decreases in the time taken to complete the TUG, and decreases in both fear of falling and depression were noted (Table 2). Qualitative data indicated that participants in all samples regarded the dance intervention favourably, noting gains to overall health and well-being. Overall, the data showed that the dance programme appeared acceptable to participants, feasible to implement and could potentially offer a way to modify physical and psychosocial risk factors of falling in community dwelling adults.

This pilot data demonstrates the acceptability of using a contemporary dance programme to modify physical and psychosocial risk factors of falling. The attrition rate of 15% is similar to that reported for previous fall interventions in community dwelling older adults. Overall, median attrition rates for 44 studies that examined fall interventions in community dwelling individuals range from 9.1–16.0% [50], and for those programmes that were either exercise alone or multifactorial where exercise was a component of the intervention, median attrition rates were 10.1% and 16% respectively. However, it is important to note that attrition rate for this review was calculated at 12 months, which is some time longer than the current 8 week study.

Acceptability was also examined via adherence rate [24, 45, 47, 51]. An adherence rate of 84.3% was noted which is similar to those which have been previously reported for other community based intervention programmes for falls [50], and either slightly less (e.g. 91%, [24]) or more (78%, [52]) for other dance related programmes. However, again, these programmes were longer in duration than the current intervention. Interestingly, our adherence rate of 84.3% was only slightly less than that recorded for a tai chi programme designed to reduce falls (88%, [53]) and while the sample size was smaller than ours (*N* = 11, vs *N* = 22), the programme was 12 weeks long, resulting in the data being more comparable to ours.

The dance programme had positive effects on both physical and psychosocial risk factors for falls [2]. Data from the IPAQ suggests that both moderate and vigorous levels of physical activity increased between T1 and T2 by 62.5 and 17.2 min respectively (see Table 2), while sitting time in the weekday decreased by 105.2 min. However, a closer look (Table 3) revealed that increases in moderate physical activity were noted by 13 of the 22 participants and the difference was probably driven by participants at site 1. While only 4 participants increased their self-reported levels of vigorous activity and in the main these were from site 3. With respect to sitting time, 7 participants reported decreases in sitting time during the week, but decreases in this measure were noted at each site (Tables 2 and 3). It may be that the reported increase in moderate and vigorous physical activity, and decrease in sedentary behaviour merely reflects the introduction of the dance and participation in the dance programme itself did not encourage further engagement in physical activity.

While the IPAQ has been shown to have fair validity and acceptable reliability in a wide range of adult populations [37], there is limited research that has investigated its applicability to elderly populations (e.g. 65+ years; [54]), with a few studies showing moderate to good reliability and validity of the IPAQ in this population [55–57]. Together with the small sample size, and lack of control group, this suggests the observed patterns of sedentary behaviour and physical activity should be interpreted with caution, and should merely serve as a means to understand the acceptability of the dance programme, rather than its effectiveness. Further work should consider more rigorous evaluation methods, such as using accelerometers to better assess changes in sedentary behaviour and physical activity and employing a treatment-as-usual control group.

Participants in the present study showed an improvement in TUG scores following the dance intervention (see Table 2). A meta-analysis has shown that older adults aged 70–79 years, which corresponds to the average age of our group (74.8 yrs), should perform the TUG test in 9.2 s (range = 8.2–10.2, [58]). Our participants’ mean TUG score at T1 was 10.1 s, which is close to the borderline of the normal range. After the dance programme the TUG score was 7.7 s which is comparable to the lower end of the range for 60 to 69 year olds [58]. The changes ranged from 0.8 s at site 3, to 3.6 and 2.5 s at site 2 and 1 respectively, with the overall change being 2.4 s (see Table 2). At an individual level (Table 3) over 80% of participants showed a decrease in their TUG time after the 8 week of contemporary dance. Interestingly, people who have danced habitually over their lives are known to have better balance than non-dancers [59, 60]. In addition, dance-based balance training or dance itself has been shown to be successful in improving balance in elderly individuals [23, 24, 61].

It is likely that the improvements in the TUG scores are a result of the nature of the dance, in that where possible a large proportion of the class is spent stepping, turning, and standing which are all important component of the TUG test. While it was not directly tested, the dance also involves contracting and exercising muscles of the legs, and it could be that the dance enhanced muscle strength of the lower limbs, which is known to plays an important role in maintaining postural control [62]. The aim of the study was not concerned with understanding the mechanisms which led to changes in balance and mobility, moreover, if dance could modify physical factors associated with falling. Given that both muscle weakness [7], and deficits in balance [8, 9] have been purported to underpin a predisposition to falls, the data from the TUG test suggests that contemporary dance could help improve balance, either direct or indirectly.

The dance programme also had a positive effect on both mood and fear of falling; two psychosocial risk factors for falls (see Table 2). In the present study only 5 participants would be considered as potentially ‘depressed’ at T1, with only 1 being so at T2. Given the baseline scores of the participants, is it not surprising that 11 people did not change on this measure, and only 6 noted mood improvements (i.e. scored lower; Table 3). Irrespective of the small number of individual changes on the GDS, there was still a significant difference between scores at T1 an T2, supporting previous research that has suggested dance has the potential to improve mental health [23, 25, 26].

Exercise interventions are generally associated with a small to moderate reduction in fear of falling in community-living older adults immediately post-intervention [63], and data from the present study also shows a contemporary dance programme has the potential to also reduce fear of falling in community dwelling adults. Fear of falling scores decreased by nearly 4 points in the present study (Table 2), with 65% of participants scoring less on the FES-I measure at T2 compared to T1 (see Table 3). Recent work has noted independent associations between decreased muscle mass, strength, power, and physical performance, and increased fear of falling [64] and research has also revealed that quadriceps strength independently contributed to explain perceived fall risk in community-dwelling [65]. It could thus be hypothesized that the additional physical activity or effects of dance on balance and muscle strength have driven the significant decrease in fear of falling in the present study. What is important is that finding ways of reducing fear of falling is vital to not only prevent falls, but to also prevent frailty. Many older adults with a fear of falling also have the potential to avoid mobility tasks, which may generate serious long-term negative effects such as physical deconditioning, muscle atrophy, and reduced social participation, which ultimately result in frailty and lack of societal independence [13].

The focus groups also highlighted other aspects of health and well-being that may have changed due to participation in the dance programme. For example, many participants noted they were practising their steps at home or without actually physically completing them, and were having to ‘think’ through the moves. Dance requires the individual to plan, monitor and execute a sequence of actions making use of a variety of executive functions, and has been suggested to benefit cognitive health in ageing [32]. Recent work has shown that approximately 6 months of contemporary dance, can indeed positively affect an individual’s capacity to switch attention between different tasks [33]. Our study suggests that aspects of cognitive health may change as a result of a CD programme and future work should examine this more closely.

While our initial data set seems promising, this study is not without its limitations. First of all while the dance programme was open to both sexes, participants were almost exclusively females (see Table 1), and the benefits of contemporary dance on risk factors for falls may only be true for women. Recent work suggests that there is an increasing need for health promotion strategies that effectively target men, that specifically focus on masculine ideals [66], and it is possible that this is not well suited to contemporary dance, thus further work is required to make this intervention attractive to both genders.

Furthermore, while this study recruited individuals who were not already part of a falls programme that was specifically designed to prevent another fall, this did not preclude those individuals who had had a fall but who had chosen to not attend a fall prevention programme offered to them by the NHS. In future, work should clearly identify those individuals who have suffered a fall, as the effect of contemporary dance could be even more beneficial.

While recruitment to the dance programme was not problematic, except at site 3 where one session of dance clashed with an existing activity, we believe this was due to the role the neighbourhood network coordinator played in supporting the recruitment process by acting as a trusted independent member of the team, with whom potential participants could discuss the project with. This approach combined with using a place-base strategy e.g. an existing community network ensured that all levels of ecological influence were accounted for to support recruitment [67]. Interestingly, despite best efforts only 22 of those that attended the dance sessions consent to complete the research element of the dance programme. Previous work has shown that telephone contact with a research assistant after receiving study information increased recruitment [68] and this should be considered in future studies trying to recruit older adults to community activity programmes.

While our initial data is promising, it must be viewed with some caution given our small sample size. Larger sample sizes and inclusion of randomised control groups would clarify changes due to dancing and be able to highlight consistency or change in measures even with high baseline scores. Furthermore, this would allow easier comparison across dance studies as well as to existing studies of Tai Chi, which seems to be one activity that research supports as being able to reduce the risk of falling [16].

While we strongly recommend larger samples, with longer term programmes and follow up, our pilot attrition and adherence data suggests that our contemporary dance programme is as acceptable as previous community based falls programmes. However, the focus groups also highlighted aspects of the programme that could have resulted in sub-optimal acceptability, and these should be considered in further iterations of the dance programme. For example, location and cost were seen both as barriers to participation as well as facilitators. Reduced mobility combined with the inability to continue driving can make accessing research institutions difficult, and transport is well recognised barrier to recruitment [69]. The focus groups also noted ensuring the balance between the creative and more didactic, choreographer led movements was very important. For some, with ongoing health problems the creative element was problematic, and they felt that they need to build up their repertoire of basic movements before engaging in more creative elements. For some however, the creative elements broke down barriers with people they didn’t know. So, while both creative and didactic elements of the programme were valued, the balance and timing of their introduction is very important, and perhaps dependent on the group dynamics and individuals’ movement capabilities.

## Conclusions

Results from this small early phase pilot study indicate a contemporary dance programme designed to modify both physical and psychosocial risk factors of falls was generally acceptable among female community dwelling older adults. The dance programme increased physical activity levels, decreased sedentary behaviour during the week, and yielded improvements in balance. In addition, the mood of those who participated in the contemporary dance programme improved and their levels of fear of falling decreased. Focus groups with participants suggest measures of quality of life and cognition should also be taken, and care should be given to the cost and location of the activity, as well as the balance between creative and didactic elements of the programme.

## Abbreviations

FES-I: Falls efficacy scale-international
GDS: Geriatric depression scale
IPAQ: International physical activity questionnaire
MPA: Moderate physical activity
NICE: National institute of clinical evidence
SWe: Sitting weekday
SWe/d: Sitting weekend
TUG: Timed up and go
VPA: Vigorous physical activity
